# Parenting by mothers from marginalized communities and the role of socioeconomic disadvantage: insights from marginalized Roma communities in Slovakia

**DOI:** 10.3389/fpsyg.2024.1362179

**Published:** 2024-04-05

**Authors:** Stanislava Van Laer, Daniela Fiľakovská Bobáková, Peter Kolarcik, Ofer Engel, Andrea Madarasová Gecková, Sijmen A. Reijneveld, Marlou L. A. de Kroon

**Affiliations:** ^1^Department of Health Psychology and Research Methodology, Faculty of Medicine, Pavol Jozef Šafárik University, Kosice, Slovakia; ^2^Graduate School Kosice Institute for Society and Health, Faculty of Medicine, Pavol Jozef Šafárik University, Kosice, Slovakia; ^3^Olomouc University Social Health Institute, Palacky University Olomouc, Olomouc, Czechia; ^4^Department of Community and Occupational Medicine, University Medical Center Groningen, University of Groningen, Groningen, Netherlands; ^5^Institute of Applied Psychology, Faculty of Social and Economic Sciences, Comenius University, Bratislava, Slovakia; ^6^Department of Public Health and Primary Care, Environment and Health, Youth Health Care, University of Leuven, KU Leuven, Leuven, Belgium

**Keywords:** parenting, mothers, marginalized Roma community, socioeconomic disadvantage, stress and worries, early childhood

## Abstract

**Background:**

Roma living in marginalized communities often face poor living conditions and material deprivation, which may negatively impact parenting. Our aim is to compare the parenting behavior (support, harsh discipline, and stimulation) of mothers from marginalized Roma communities and the majority population in Slovakia. We also examine the role of socioeconomic disadvantage and related worries in the differences in parenting behavior between these groups.

**Methods:**

We obtained cross-sectional data from mothers of children aged 14–18 months using the first wave of the longitudinal RomaREACH study dataset. Two groups were included in the sample: 93 mothers from MRCs and 102 mothers from the majority. We performed multiple regression and mediation analyses to assess whether the educational level of mothers, the degree of poverty, and poverty-related feelings of stress and worries explain parenting behavior differences between the groups of mothers.

**Results:**

We found significant differences in parenting, especially in harsh disciplining and stimulation. These two domains were significantly associated with maternal education, degree of poverty, and poverty-related stress and worries. The degree of poverty partially mediated stimulation differences between the two groups of mothers.

**Conclusion:**

Parenting in MRCs seems harsher and less stimulative than parenting in the Slovak majority. These differences are associated with the socioeconomic disadvantage of mothers. The degree of poverty partially explains why parenting in MRCs is less stimulative. These results may inform intervention efforts aimed at disadvantaged families.

## 1 Introduction

The experience of motherhood is very diverse and influenced by structural factors such as social exclusion, marginalization, and discrimination. Such unfavorable circumstances affect how women with different ethnic backgrounds from disadvantaged groups constitute their motherhood and raise their children in the context of poverty and stress (Čanigová and Souralová, 2024). The association of poverty with parenting practices and their effect on early childhood development is well documented across countries worldwide (Tran et al., 2017). Responsive parenting considered to be the optimal approach across cultures (Ekmeci et al., 2016). It is defined by characteristics such as attention to the child, relevance and predictability of response, or sufficient socio-emotional support provided to the child present in every parent–child interaction (Cortés-Moreno, 2017). The quality of parenting can be negatively affected by stress stemming from both a disadvantaged socioeconomic status and ethnic minority status (Nomaguchi and Milkie, 2020).

The Roma are one of the largest disadvantaged populations in Europe (European Union Agency for Fundamental Rights, 2022). A major contributor to this disadvantage regards anti-Roma racism (Alexiadou, 2023) perpetuated through a stereotypical, racist, and discriminatory discourse, which hampers the successful inclusion of Roma (Izsak-Ndiaye, 2015). Roma across the EU continue to face high levels of discrimination in the education and labor market, in healthcare and in other public or private services based on their origin (European Union Agency for Fundamental Rights, 2022). Accordingly, they are among the most discriminated groups in Europe. According to the Pew Research Center (2019), more than three-quarters of people in Slovakia hold negative views of Roma, which is more than in the neighboring countries. However, also in these countries, such views were found to be common for 50–60% of the respondents.

The Roma minority often faces poor living conditions and material deprivation, particularly in marginalized communities. It is estimated that more than half out of 440,000 Roma in Slovakia live in marginalized Roma communities (MRCs) (Atlas of Roma Communities in Slovakia, 2019). Recent data show that 87% of households in MRCs are at risk of poverty and more than half face poor housing conditions and serious material deprivation (Markovic, 2020). This extremely unfavorable socioeconomic situation (regardless of ethnicity) can highly influence the family environment toward greater instability in daily family routines and higher levels of chaos (Evans, 2004). Material hardship such as food insecurity, residential instability or financial troubles can lead to marital conflict, parenting stress and depressive symptoms, which may impact parenting practices and further fuel a downward cycle impacting the healthy development of children (Gershoff et al., 2007). In the first 1,000 days of life, the rapid process of neurodevelopment takes place, and the foundation is laid for all future learning, behavior, and health of a human being (Campbell et al., 2014). Child poverty may have a detrimental effect on children from MRCs who are exposed to many early life stressors.

Extensive evidence shows supportive and stimulating caregiving can promote children’s developmental outcomes (McCartney et al., 2007). Parental support manifests as showing affection, involvement, acceptance, emotional availability, warmth, and responsivity to the child (Cummings et al., 2000). Parents form a reliable base from which children can explore and safely return for reassurance when encountering difficulties (Verhoeven et al., 2010). They can provide an engaging environment and opportunities where children can learn and develop (Urke et al., 2018). On the other hand, children’s development and regulatory capacities can be undermined by harsh disciplining, such as physical punishment, yelling, criticism, and controlling children’s behavior (Baydar and Akcinar, 2018). Parental influence seems vital to the child’s development (Belsky and de Haan, 2011; Tost et al., 2015).

Research on the quality of parenting behavior in MRCs is very scarce. The limited evidence shows that parenting in Roma families is primarily a task of the mother and the oldest siblings, but that also wider relatives and the whole community participate in the upbringing of their children (Šebková, 1996; Žigová, 1996; Smith, 1997). The parenting approach in Roma families is less restrictive than the parenting approach in the majority population. Children are often left to explore the world on their own and experiment and learn by observing the usual daily activities of their community (Smith, 1997). Mothers from MRCs in Slovakia seem to prefer responsive parenting, which can be described as noticing and acting on children’s interests, speech, and nonverbal communication, as an optimal parental approach (Chovan, 2019). However, evidence to what extent Roma mothers can live up to their parental ideals is non-existent.

Gaining insight into the parenting behavior of mothers from MRCs may inform evidence-based early interventions favoring an adaptive child development of marginalized Roma children. Understanding the differences in parenting behaviors and recognizing the interplay between poverty, stress and parenting practices might help to change unfavorable public and political discourses and help to design and target interventions. Interventions that address the elementary needs of the families in MRCs or support parents’ knowledge and skills in providing a nurturing environment for their children can draw on this evidence to prioritize and achieve the greatest possible synergistic effect. Therefore, we undertook the longitudinal RomaREACH study on early childhood, which is one of the first to examine parenting in MRCs and address selected psychological and socio-cultural aspects of the processes shaping parenting quality and child development.

Our aim is to compare the parenting behavior (support, harsh discipline, and stimulation) of mothers from marginalized Roma communities and the majority population in Slovakia. We also examine the role of socioeconomic disadvantage and related worries in the differences in parenting behavior between these groups.

## 2 Materials and methods

### 2.1 Sample and setting

We used cross-sectional data from the first wave of the longitudinal RomaREACH study. The data were collected in 2021–2022 in Slovakia’s Prešov and Košice regions. Our sample consisted of 195 mothers of children aged 14–18 months. We chose this age because all children in Slovakia are invited for a mandatory preventive check-up at this age. Also, around this age, children typically enter a phase of rapid development of complex skills such as walking, talking, and problem-solving; progress can be seen in social interaction and emotional regulation, which enables to assess larger differences between children than at younger ages. Moreover, at this age, the influence of parenting becomes clearer since the quality of interactions with caregivers and opportunities for exploration and learning highly influence development (Benson, 2020). We focused on children and their mothers as mothers (especially in MRCs) are primary caregivers, and fathers who are involved in caregiving with infants of such a young age to a much lesser extent (Obrovská and Sidiropulu Janků, 2021; Čvorović, 2022) are less willing to participate in family research (Voicu and Popescu, 2009).

We included two groups of mothers in the sample: 93 mothers were from marginalized Roma communities (MRCs), and 102 mothers were from the Slovak majority population (general population of mostly middle-to-high socioeconomic status). Participants were recruited in three ways: (1) via pediatricians during regular preventive check-ups (mothers from both populations), (2) via Roma health mediators and social workers directly in the communities (mothers from MRCs), (3) via parental groups on social media (mothers from the majority). The primary recruitment route was via pediatricians during mandatory preventive check-ups. However, due to a crisis in primary pediatric care induced during the COVID-19 pandemic, which led to a work overload of pediatricians and thus less room to invest in recruitment, we also employed other recruitment routes. Using Roma health mediators and social workers who have the trust of community members as intermediaries for recruitment enabled us to reach a sufficient sample size, which would not have been possible otherwise, especially considering that the population living in MRCs is challenging to engage and thus underrepresented in the research.

The data were collected through a self-report questionnaire. The data collection took place either in the cooperating outpatient departments, in the community centers, or the households of mothers from medium to high socioeconomic status Slovak majority population. Mothers filled out paper questionnaires independently or with the assistance of researchers. We used assisted self-administered interviews to cope with the low literacy of mothers from MRCs. This regards a modification of other methods of collecting survey data, which has been shown to lead to data with good validity (Tourangeau and Smith, 1996). We obtained signed informed consent from all participating mothers. Participation in the study was entirely voluntary and confidential. The Roma REACH study was approved by the Ethics Committees in both the Prešov and the Košice regions and by the Ethics Committee of the Medical Faculty at P.J. Šafárik University in Košice under No. 03682/2022/OZ-20, “RomaREACH,” and 16 N/2021, respectively.

### 2.2 Measures

We assessed parenting, sociodemographic variables, and poverty-related feelings of stress and worries of mothers from marginalized Roma communities and mothers from the Slovak majority. To assess the degree of poverty, we included relatively objective indicators, such as maternal education and availability of water and electricity in the household, but also a subjective indicator, i.e., the degree to which everyday struggles of people living in poverty translate into feelings of stress. These measures were developed in close cooperation with Roma health mediators who have intimate knowledge of the context of MRCs and used in other studies with MRCs enabling to assess the degree of poverty on the lower socioeconomic spectrum, which is not possible using only educational level as an indicator (Gecková et al., 2014; Belák, 2020). Before the data collection, measures were tested in a pilot study, which included 405 mother–child dyads from MRCs and the majority population. A subsample of 30 mothers was interviewed about problematic items they found challenging to answer or understand. Qualitative and statistical analyses were performed. Necessary adaptations were made to increase the understandability of problematic items while preserving their meaning.

#### 2.2.1 Parenting

Parenting was assessed using three domains of the comprehensive early childhood parenting questionnaire (CECPAQ), which covers five domains of parenting: i.e. support, harsh discipline, stimulation, structure, and positive discipline (Verhoeven et al., 2016). The CECPAQ was developed in the Netherlands to measure the parenting behavior of parents with children aged one to five, i.e., it covered our age group of interest. The questionnaire showed good psychometric characteristics (Verhoeven et al., 2016). The tool is currently used in diverse settings across and outside Europe. For our study aims, we excluded the domains Positive discipline and Consistency, which contain items asking about parental approaches applicable to older children of around 3 years of age with sufficient cognitive skills and self-regulation to understand and process rules and their explanations or consequences of certain behaviors (Montroy et al., 2016). We thus used the domains of the CECPAQ that are relevant for the optimal development of children in the targeted age range of 14–18 months, i.e., (1) support (13 items, Cronbach’s α = 0.81), which includes data on sensitivity, responsiveness, and affection, (2) harsh discipline (12 items, Cronbach’s α = 0.90), which includes data on verbal, physical, and psychological control, and (3) stimulation (13 items, Cronbach’s α = 0.83), which includes data on activities, exposure, and toys. The response categories indicated how often parents showed the described behavior: (1) never/does not concern me, (2) very rarely, (3) rarely, (4) occasionally, (5) very often, (6) always. Sum-scores ranged from 0 to 65 in support and stimulation and from 0 to 60 in harsh discipline. A higher score indicates more support and stimulation and more frequent use of harsh discipline practices.

#### 2.2.2 Sociodemographic variables

The sociodemographic variables included in the study were the educational level of mothers and the availability of water and electricity in the household as a proxy variable of the degree of poverty.

The level of maternal education (Kolarcik et al., 2009) was assessed by the question: “What education did you complete? (Elementary school, Apprentice school, Secondary school, University).” We categorized mothers’ education into three categories: (1) elementary education, (2) secondary education, including apprentice school, and (3) university education.

We assessed the degree of poverty by determining the availability of water and electricity in the household using the question: “Is the following in your household? (Running water, Electricity)” (Gecková et al., 2014). The response categories were “Yes” and “No.” The households with the availability of both were categorized as: water and electricity available in the household. The households missing either water or electricity or both were categorized as: having no water and/or no electricity in the household.

#### 2.2.3 Poverty-related feelings of stress and worries

We measured the Poverty-related feelings of stress and worries (Belák, 2020) by asking the questions: “Have you been worried/experienced stress about the following in the past 3 months?” (10 items, Cronbach’s *α* = 0.79) (see Appendix 1 for the topics that might generate feelings of stress and worries used in the questionnaire). The response categories for each topic were “Yes” and “No.” We created a sum score for each participant, i.e., a sum of the ‘Yes’ answers provided for each question, with a higher score indicating more poverty-related feelings of stress and worries. This sum-score was categorized as: (1) no worries, (2) one to two worries, and (3) three or more worries.

### 2.3 Statistical analyses

First, we described the sample using descriptive statistics. Differences between the two groups (mothers from marginalized Roma communities vs. mothers from the majority) were assessed using non-parametric Mann–Whitney *U*-tests for continuous variables which were not normally distributed and Chi-square tests for dichotomous variables. Second, we performed simple regression analyses (Model 1) to assess the relationship between belonging to one group and parenting domains ‘support’, ‘harsh discipline’, and ‘stimulation’. After that, we performed multiple regression analyses adjusting for infants’ sex and maternal age (Model 2) as these might influence the relationships as found (Camberis et al., 2016; Morawska, 2020). We created dummy variables for elementary and secondary education with university education as a reference category. Next, we performed multiple regression analyses additionally for maternal education, availability of water and electricity in the household, and poverty-related feelings of stress and worries (Model 3) to assess the contribution of these indicators of disadvantage to the differences in parenting between mothers from MRCs and the majority population. We computed bootstrapped estimates and confidence intervals (CIs) for the support and the harsh parenting domains, as the residuals of these models were not normally distributed. Third, we conducted mediation analyses to assess whether the educational level of mothers, the degree of poverty, and poverty-related feelings of stress and worries mediated the differences in parenting behavior between the two groups. SPSS 23 for Windows was used for the first two steps, and the R Package for Causal Mediation Analysis (Tingley et al., 2014) was used for the third one.

## 3 Results

### 3.1 Description of the sample

**Figure 1:**
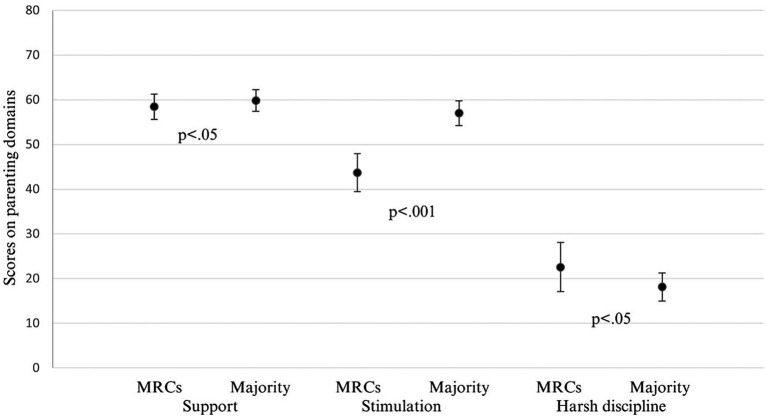
Comparisons of the mean scores (Mann–Whitney *U* test) and standard deviations for support, stimulation, and harsh discipline in mothers from marginalized Roma communities (MRCs) and mothers from the majority population in Slovakia.

Table 1 and Figure 1 show the descriptive statistics for mothers from marginalized Roma communities and mothers from the majority population. A few significant differences between the groups were found. In MRCs, compared to the majority population, the maternal age and education were lower. In contrast, the number of children in the household and poverty-related feelings of stress and worries were higher in MRCs. Access to water and electricity was less available in MRCs compared to the households of mothers from the majority. Significant differences were also found between the two groups in all three parenting domains, with mothers from the majority scoring higher in support and stimulation and mothers from MRCs scoring higher in harsh discipline.

**Table 1:** Description of the studied groups: mothers from the marginalized Roma communities vs. mothers from the majority population in Slovakia.

	Marginalized Roma communities (*N* = 93)		Majority population (*N* = 102)		Total (*N* = 195)		*p*-value*
*Sex*							
Girls (N, %)	48	51.6	58	56.9	106	54.4	0.46^a^
Boys (N, %)	45	48.4	44	43.1	89	45.6	
Age of children in months (mean, SD)	15.91 (1.21)		15.87 (1.07)		15.89 (1.14)		0.76^b^
Age of mothers in years (mean, SD)	25.24 (5.82)		32.05 (4.46)		28.8 (6.17)		< 0.001^b^ *
Number of children in household (mean, SD)	4.10 (2.76)		1.63 (0.73)		2.81 (2.33)		< 0.001^b^ *
Maternal education							< 0.001^a^ *
Elementary (N, %)	73	78.5	2	2.0	75	38.5	
Secondary (N, %)	20	21.5	21	20.6	41	21.0	
University (N, %)	0	0.0	79	77.5	79	40.5	
Water & electricity^c^							< 0.001^a^ *
No (N, %)	49	53.3	2	2.0	51	26.3	
Yes (N, %)	43	46.7	100	98.0	143	73.7	
Poverty-related feelings of stress and worries (mean, SD)^c^	2.27 (2.37)		0.48 (0.90)		1.32 (1.96)		< 0.001^b^ *

^*^Statistically significant differences between Roma and non-Roma.

^a^ Chi-square test.

^b^ Mann–Whitney *U* test.

^c^ Missing values; for MRC for Water & electricity: 1; poverty-related feelings of stress and worries: 3.

### 3.2 Differences between mothers from marginalized Roma communities and the majority

Table 2 shows the results of the linear regression analyses. In all three parenting domains, mothers from marginalized Roma communities and mothers from the majority population differed significantly (Model 1; Figure 1). Mothers from MRCs reported less support and stimulation and more use of harsh discipline practices compared to mothers from the majority population. After adjusting for sex and maternal age (Model 2), the relationships remained statistically significant for harsh discipline and stimulation.

**Table 2:** Comparison of parenting (support, harsh discipline, and stimulation) of mothers from marginalized Roma communities (MRCs) with that of the majority population (‘majority’) in Slovakia, crude and adjusted for infant sex and maternal age using simple and multiple linear regression analyses.

	Model 1 (crude) B (95% CI)	Model 2 (adjusted for infant sex and maternal age) B (95% CI)	Model 3 (additionally adjusted for socioeconomic disadvantage # and worries) B (95% CI)
*Support*			
MRCs vs. majority	−1.66 (−3.19; −0.24)*	−1.23 (−3.06; 0.62)	−2.24 (−5.62; 0.92)
Sex	−0.36 (−1.82; 1.11)	−0.43 (−1.85; 1.16)	−0.58 (−2.20; 1.01)
Maternal age in years	0.12 (0.00; 0.24)*	0.07 (−0.07; 0.22)	0.07 (−0.08; 0.22)
*Maternal education (ref: university)*			
Elementary	−0.98 (−2.47; 0.52)		1.48 (−2.20; 4.68)
Secondary	−0.44 (−2.23; 1.35)		0.32 (−2.00; 2.27)
Water & electricity available	0.69 (−0.96; 2.33)		−1.03 (−3.34; 1.10)
Poverty-related feelings of stress and worries	−0.31 (−0.69; 0.06)		−0.30 (−0.82; 0.15)
Harsh discipline			
MRCs vs. majority	4.43 (1.94; 6.83)***	4.24 (1.16; 7.16)**	2.02 (−2.93; 7.05)
Sex	−0.16 (−2.63; 2.30)	0.42 (−1.93; 2.67)	0.47 (−2.09; 2.82)
Maternal age in years	−0.25 (−0.45; −0.05)*	−0.05 (−0.29; 0.21)	−0.06 (−0.31; 0.21)
*Maternal education (ref: university)*			
Elementary	4.93 (2.49; 7.37)***		2.70 (−2.94; 7.67)
Secondary	−1.02 (−4.01; 1.98)		0.25 (−2.16; 2.71)
Water & electricity available	−3.05 (−5.85; −0.26)*		0.66 (−4.08; 5.25)
Poverty-related feelings of stress and worries	0.90 (0.27; 1.52)**		0.30 (−0.63; 1.32)
*Stimulation*			
MRCs vs. majority	−13.18 (−15.13; −11.22)***	−14.33 (−16.76; −11.90)***	−9.28 (−13.64; −4.92)***
Sex	0.20 (−2.53; 2.93)	−0.59 (−2.57; 1.39)	−0.40 (−2.36; 1.56)
Maternal age in years	0.56 (0.35; 0.77)***	−0.13 (−0.33; 0.71)	−0.13 (−0.33; 0.07)
*Maternal education (ref: university)*			
Elementary	−11.41 (−13.68; −9.14)***		−3.83 (−8.49; 0.84)
Secondary	−0.31 (−3.65; 3.02)		−2.07 (−5.36; 1.21)
Water & electricity available	11.57 (8.97; 14.17)***		3.57 (0.66; 6.49)*
Poverty-related feelings of stress and worries	−1.76 (−2.40; −1.12)***		0.06 (−0.53; 0.65)

*p < 0.05; **p < 0.01; ***p < 0.001.

#socioeconomic disadvantage regards maternal education, availability of water and electricity in the household and poverty-related feelings of stress and worries.

Adding maternal education, availability of water and electricity in the household, and poverty-related feelings of stress and worries to Model 2 further decreased the strength of the relationships for all three parenting domains and left stimulation as the only domain with a significant association. In the presence of other covariates in Model 3, the availability of water and electricity was significantly associated with stimulation only (see Model 3).

### 3.3 Mediation analyses

Out of the three studied socioeconomic disadvantage-related variables (maternal educational level, availability of water and electricity in the household, and poverty-related feelings of stress and worries), none were significantly associated with support. As for stimulation, all three socioeconomic disadvantage-related variables were significantly associated: elementary maternal education (B/CI: −11.41/−13.68; −9.14), availability of water and electricity in the household (11.57/8.97; 14.17), and poverty-related feelings of stress and worries (−1.76/−2.40; −1.12). The same was the case for harsh disciplining: elementary maternal education (4.93/2.49; 7.37), availability of water and electricity (−3.05/−5.85; −0.26), and poverty-related feelings of stress and worries (0.90/0.27; 1.52).

**Figure 2:**
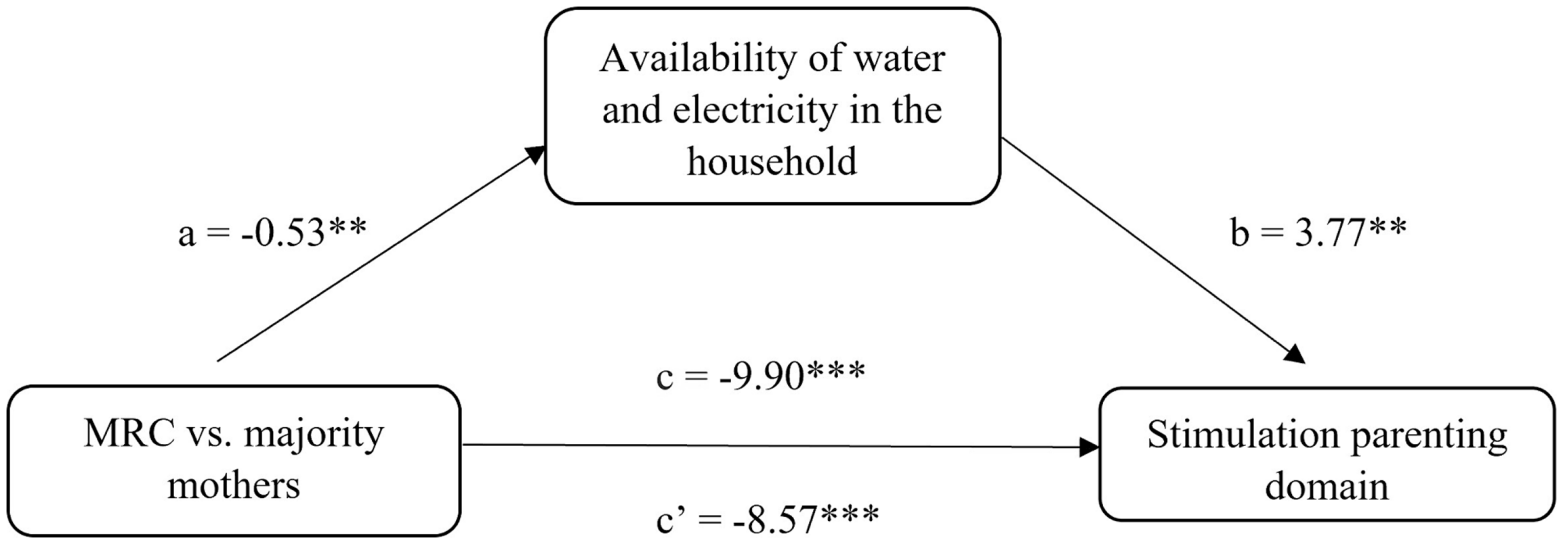
The mediation effect of availability of water and electricity in the household in the association between MRC vs. majority mothers and stimulation in parenting. *p < 0.05; **p < 0.01; ***p < 0.001.

Notes: *a* is the effect of MRC vs. majority mothers on the availability of water and electricity in the household, *b* is the effect of availability of water and electricity in the household on the stimulation parenting domain, *c’* is the direct effect of MRC vs. majority mothers on the stimulation parenting domain and *c* is the total effect of MRC vs. majority mothers on the stimulation parenting domain.

Next, we found only one mediation effect. The effect of belonging to the marginalized Roma communities vs. belonging to the majority population on the outcome of stimulation in parenting was mediated by the availability of water and electricity in the household. This model (Figure 2) showed a significant direct effect (−8.57/−12.63; −4.17) and a significant indirect effect (−1.32/−3.04; −0.10), both contributing to a significant total effect of the exposure on the outcome (−9.90/−14.16; −5.72).

## 4 Discussion

Our study aimed to compare the parenting behavior (support, harsh discipline, and stimulation) of mothers from marginalized Roma communities and the majority population in Slovakia and examine the role of socioeconomic disadvantage and related worries in the differences in parenting behavior between these groups. We found significant differences in parenting between the two populations, especially in harsh disciplining and stimulation. We also found that the degree of poverty (availability of water and electricity in the household) partially explained the differences in stimulation in parenting.

First, our findings indicate that mothers from MRCs use harsh discipline practices (verbal, physical, and psychological control) more frequently compared to mothers from the majority population and that lower education, higher degree of poverty, and the number of poverty-related feelings of stress and worries are significantly associated with more frequent use of harsh disciplining. These findings align with previous research. Numerous researchers confirm that parents reporting higher levels of stress use more punitive and control-oriented exchanges with their children (Conger and Donnellan, 2007; Roubinov and Boyce, 2017) and that additional years of maternal schooling reduce the usage of harsh parenting practices (Jansen et al., 2012; Cuartas, 2022). Financial hardship concerning harsh disciplining was studied, for example, by Anderson et al. (2022), Jansen et al. (2012), Ho et al. (2022), and Respler-Herman et al. (2011), confirming its contribution to harsh parenting. Our results suggest that the level of harsh parenting mothers engage in is related to their socioeconomic disadvantage and related worries.

Second, we found that mothers from MRCs stimulated their children less than mothers from the majority with the included sociodemographic variables (maternal education, degree of poverty) and the number of poverty-related feelings of stress and worries being significantly associated with stimulation. Our results align with previous research confirming that highly educated mothers invest more time in basic care and play with their children than less-educated mothers (McLanahan, 2004). Low income, typically associated with poverty, is also proven to be related to poorer involvement of parents in children’s play activities (Kalil and Ryan, 2020). The experience of poverty seems to be linked to the lack of stimulation via parental emotional distress (Iruka et al., 2012). When it comes to belonging to the group (MRC vs. majority) being a predictor of stimulation, the explanation may be that ethnic minority mothers do not only experience heightened stress related to their socioeconomic status but also experience stressors specific to their minority status, which has also been previously found to affect parenting quality negatively (Leidy et al., 2010; Nomaguchi and Milkie, 2020). The level of maternal involvement in interacting with their children in a stimulating way depends on their educational level, the degree of poverty, and the amount of stress and worries related to the poverty they experience. The differences in how mothers stimulate their children also seem to have their origin in the group a mother belongs to, MRC or majority.

Third, we found that support differed little between mothers from MRCs and mothers from the majority, and the differences disappeared when adjusting for the lower age of Roma mothers. The findings on the role of maternal age as a significant predictor of the level of support confirm previous research. For example, Camberis et al. (2016) found that older mothers are more psychologically mature and that this maturity indirectly contributes to more sensitive and mind-minded interactions between the mother and her infant. At the same time, the environment created by poverty makes adolescent motherhood more likely (Cook and Cameron, 2020). Thus, it is likely that the younger the mother from MRC, the more challenges she faces in providing sensitive, responsive, and affectionate parenting to her children.

Finally, we found that only poverty, i.e., ‘availability of water and electricity in the household’, partly mediated the differences in stimulation between mothers from MRCs and mothers from the majority in Slovakia. Deprived parents who experience a high degree of poverty may face a range of challenges that can impact their ability to provide a nurturing environment for their children (Gershoff et al., 2007; Kalil and Ryan, 2020). Households with limited access to basic amenities cannot afford to create a more stimulating environment for their children to engage in learning activities together. However, limited access to water and electricity does not prevent mothers from being supportive or using a positive disciplinary approach. A possible explanation for poverty-related distress, not explaining the differences between the two groups of mothers in stimulation, is that stress is a highly subjective experience. Two individuals experiencing the same stressful event may appraise and thus cope with the stressful event differently (Jacoby et al., 2021). Regarding maternal education, the collinearity with the group a mother belongs to may be too high for the mediation effect to occur. It seems understandable that poverty explains the differences in stimulation between the groups of mothers since only when the most elementary needs are met can parents acquire the capacity to provide a stimulative environment for their children (Maslow, 1954). There may also be other potential mediators related to stimulation, which we did not study. Often, families in MRCs have a higher number of children. Thus, the attention of mothers must be split between more siblings who take over some of the responsibilities and engage in activities with their younger siblings simultaneously. Nevertheless, poverty partially explains less stimulating parenting in MRK, which might be related to the fact that the parental mental and material capacity to focus on their children is limited in an environment characterized by poverty, such as in MRCs.

### 4.1 Strengths and limitations

Our study, which uses cross-sectional data from the first wave of the longitudinal RomaREACH study, is one of the first to focus on mothers’ parenting in MRCs. It brings insights into how parenting in this specific context differs from that of the majority population in the same region and addresses the contribution of severely adverse socioeconomic factors using multiple regression and mediation analyses. Moreover, studies on parenting usually do not focus on parenting approaches to children at such a young age. Using CECPAQ to measure parenting allowed us to look at multiple dimensions simultaneously. Our findings confirm the adverse effect of poverty on the quality of parenting on one hand and on the other hand point to the complexity of disadvantage accumulated in the environment of MRCs, which translates into the challenges of parenthood. These findings have a potential for informing evidence-based early interventions aimed at disadvantaged families. The study’s main limitations are the size of the sample and its cross-sectional design. The limited sample size lowered the power of the study to detect all effects and differences, implying that only large differences and a strong mediation could be detected. A cross-sectional design hinders conclusive inferences about causality and long-term outcomes. Furthermore, we collected self-reported data, which may be prone to social desirability. However, we reduced the likelihood of that by providing a safe environment for the mothers when answering the questions. Data from the mothers living in MRCs were mostly collected via assisted self-administered interviews in the Slovak language, whereas data from mothers out of the majority were collected via self-reported questionnaires. We did so to cope with illiteracy, which we considered a more serious source of non-response and bias than using two different types of administration, with both types having been shown to lead to reliable and valid data (Tourangeau and Smith, 1996). Also, the challenging setting in which the study was conducted led to lower response rates and might have impacted the selection of the respondents (especially approaching mothers via social media). We were not able to include either Roma mothers with higher socioeconomic status outside of MRCs nor mothers from a majority living at a level of disadvantage comparable to that of the vast majority of mothers from MRCs included in our sample. This implies that the groups, as compared, are rather distinct and represent opposite sides of the socioeconomic spectrum. This somewhat limits the generalizability of the findings as Roma are a very heterogeneous ethnic group in terms of living conditions and levels of integration (Zachar Podolinská and Škobla, 2018). However, our findings may be representative of the subsection of the Roma population living in poverty in communities characterized by social and physical distance from the majority population; because of their novelty, they need confirmation in future studies.

### 4.2 Implications for practice, policy, and research

We found differences in harsh disciplining and stimulation, which imply a need for supporting marginalized Roma mothers to provide stimulating care to their children by having a range of respectful alternatives to harsh disciplining at hand. A good example of a culturally sensitive and effective intervention in the Slovak environment is the project Omama (Cesta von, 2023). Trained women from MRCs called Omamy regularly visit hundreds of families in their home environment. They teach mothers how to effectively stimulate their children’s development by playing with them during regular lessons. The advantage lies in employing women who speak the community’s language and live within it, thus having an in-depth knowledge of the settings and of the families they work with. We also found that maternal education, access to water and electricity in the household, and poverty-related stress and worries are all significantly associated with harsh discipline and stimulation. Supporting the education of women from MRCs, in general, can give them the tools they need to break the cycle of poverty and ultimately provide a more nurturing environment for their children. Securing access to water and electricity would undoubtedly make parenting less challenging and stressful.

The differences in stimulation between the two groups of mothers were partly mediated by the degree of poverty, i.e., the availability of water and electricity in the household. The very first step in supporting mothers in better stimulating their children should be securing access to basic amenities such as water and electricity. Only after the fundamental needs are met all other policies aimed at marginalized Roma mothers and their children can be of benefit. Further research should explore whether the differences between mothers from MRCs and mothers from a majority group also hold for other countries and settings. Moreover, enriching self-reported with observational data on parents interacting with their children may add, as well as qualitative research providing insights into how optimal parenting is perceived in MRCs and what challenges mothers face in parenting. Future research might also consider assessing the role of different social and cultural capital (Qi et al., 2023) in parenting. On top of that, adding measures on stress and worries specific to their minority status could be considered in addition to stress and worries stemming from poverty. Finally, longitudinal designs and use of biological parameters could give more insight into the causal pathways between the predictors and parenting domains.

## 5 Conclusion

We found significant differences in parenting between mothers from MRCs and mothers from the majority in Slovakia, especially in the harsh discipline and stimulation domains. Both harsh discipline and stimulation were significantly associated with the age of mothers, maternal education, degree of poverty, and the amount of poverty-related stress and worries. The degree of poverty had a mediator role, partly explaining the differences in stimulation between mothers from MRCs and mothers from the majority in Slovakia. Our results may inform intervention efforts aimed at families from disadvantaged environments to overcome the negative effects of poverty on parenting practices.

## Data availability statement

The raw data supporting the conclusions of this article will be made available by the authors, without undue reservation.

## Ethics statement

The studies involving humans were approved by Ethics Committees in both the Prešov (No. 03682/2022/OZ-20) and the Košice (“RomaREACH”) regions and by the Ethics Committee of the Medical Faculty at P.J. Šafárik University in Košice (16N/2021). The studies were conducted in accordance with the local legislation and institutional requirements. Written informed consent for participation in this study was provided by the participants’ legal guardians/next of kin.

## Author contributions

SL: Conceptualization, Data curation, Formal analysis, Investigation, Writing – original draft, Writing – review & editing. DF: Data curation, Funding acquisition, Investigation, Methodology, Project administration, Supervision, Writing – review & editing. PK: Formal analysis, Methodology, Writing – review & editing. OE: Formal analysis, Writing – review & editing. AM: Supervision, Writing – review & editing. SR: Conceptualization, Supervision, Writing – review & editing. MK: Conceptualization, Supervision, Writing – review & editing.
