# Supplementary material for: Parenting by mothers from marginalized communities and the role of socioeconomic disadvantage: insights from marginalized Roma communities in Slovakia
Source: Front Psychol. 2024 Apr 5;15:1362179. doi: 10.3389/fpsyg.2024.1362179 (PMC11026858; doi:10.3389/fpsyg.2024.1362179)
Supplement: Supplementary file 1 [file Table_1.DOCX]

‌**Appendix 1.**

**Topics used to measure poverty-related feelings of stress and worries**

Have you been worried (or under stress) about any of the following in the past three months?

(a) Worries that you will lose a roof over your head,
(b) Worries that there will not be any food to eat in the household,
(c) Worries that your children will be taken away from you,
(d) Worries that there will be consequences of not being able to pay your debts back,
(e) Worries about the cold and the lack of finances for heating,
(f) Worries about the danger of being imprisoned/having someone close being imprisoned,
(g) Worries about a seriously ill loved one,
(h) Worries or stressing out about losing a job/a close person losing a job,
(i) Stress about home violence,
(j) Stress about a hurtful physical or verbal attack or stress about the fights or other rough conflicts with people outside the household?”
